# Endogenous localization of TOP-2 in *C. elegans* using a C-terminal GFP-tag

**DOI:** 10.17912/micropub.biology.000402

**Published:** 2021-05-28

**Authors:** Christine K. Rourke, Darline Murat, Tyler J. Hansen, Aimee Jaramillo-Lambert

**Affiliations:** 1 Department of Biological Sciences, University of Delaware, Newark, Delaware 19716; 2 Currently-Department of Biochemistry, Vanderbilt University School of Medicine, Nashville, Tennessee 37205

## Abstract

To investigate the dynamic localization of Topoisomerase II in live *C. elegans* we have generated a C-terminally GFP-tagged version of TOP-2 at the endogenous locus. We found that TOP-2::GFP localizes in a similar pattern to the previously published TOP-2::3XFLAG strain and does not disrupt the meiotic chromosome segregation functions of this enzyme.

**Figure 1. Endogenous TOP-2 localization using a GFP-tag:**
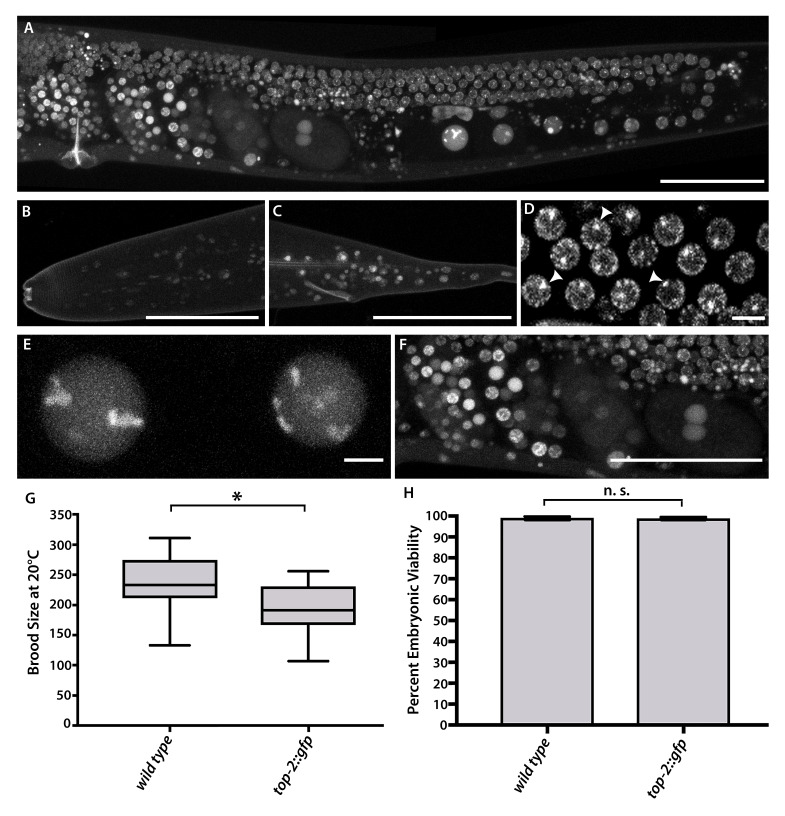
(A-F) Live imaging of the *top-2::gfp C. elegans* strain using confocal microscopy. (A) A complete representation of TOP-2 localization in the germ line of *top-2::gfp* worms. Scale bar= 50 µm. (B, C) TOP-2 localization in the head and tail, respectively, of *top-2::gfp* worms. Scale bar= 50 µm (D) TOP-2 localization during pachytene of *top-2::gfp* worms. White arrowheads point to examples of TOP-2::GFP foci. Scale bar= 5 µm. (E) TOP-2 localization in -1 and -2 oocytes in *top-2::gfp* worms. Scale bar= 5 µm. (F) TOP-2 localization in developing embryos in *top-2::gfp* worms. Scale bar= 50 µm. (G) Brood size comparison of endogenously tagged *top-2::gfp* to wild type (N2). (H) Percent embryonic viability from the *top-2::gfp* strain compared to wild type (N2). Statistics were conducted using a two-tailed Student’s T-test, n.s. indicates not significant, *p=0.0001.

## Description

Topoisomerase II is a homodimeric, ATP-dependent enzyme that is conserved across species (Dong and Berger 2007; Nitiss 2009; Kim, Lee, and Koo 2000; Pommier *et al.* 2016; Vos *et al.* 2011). Topoisomerase II (Topo II) is expressed in all eukaryotic cells and is essential for many cellular processes including replication, transcription, chromosome structure, and chromosome segregation during mitosis (Nitiss 2009). In meiosis, Topo II is required for proper homologous chromosome segregation at the first meiotic division (Jaramillo-Lambert *et al.* 2016; Rose, Thomas, and Holm 1990; Marchetti *et al.* 2001; Hughes and Hawley 2014). In *C. elegans*, the complete absence of the Topo II homolog, TOP-2, results in sterility, while a loss-of-function mutation, *top-2(it7)*, exhibits a sex-specific chromosome segregation defect at anaphase I of meiosis in spermatogenesis. This chromosome segregation defect is not observed during oogenesis (Jaramillo-Lambert *et al.* 2016). Previously, we generated a TOP-2::3XFLAG strain to visualize TOP-2 localization in dissected gonads (Jaramillo-Lambert *et al.* 2016). Probing for anti-FLAG on dissected gonads of the TOP-2::3XFLAG strain, we observed nucleoplasmic TOP-2 localization in the mitotic proliferative zone, which then becomes associated with chromosomes during the transition zone, pachytene, and diplotene regions of the germ line. During late meiotic prophase I (diakinesis), TOP-2 completely dissociates from the chromosomes leaving a nucleoplasmic pool in the oocytes. In order to visualize the dynamic localization of TOP-2 during meiosis in *C. elegans* using live imaging, we created a TOP-2::GFP tagged strain via CRISPR/Cas9 genome editing at the endogenous *top-2* locus. The endogenous C-terminal TOP-2::GFP is expressed in nuclei throughout the worm soma, germ line, and in developing embryos (Figure 1A-F). In the germ line, TOP-2 is expressed from the mitotic zone at the distal tip to the most proximal oocyte, which is preparing for fertilization (Figure 1A). During pachytene, TOP-2 is localized to the chromosomes (Figure 1D) similar to that observed in the anti-FLAG staining of TOP-2::3XFLAG germ lines. Interestingly, we observed a single TOP-2::GFP focus in pachytene nuclei (Figure 1D, white arrowheads). These foci are not observed with the anti-FLAG antibody staining in the TOP-2::3XFLAG germ lines. In contrast to the strictly nucleoplasmic pool of TOP-2::3XFLAG during late meiotic prophase I (diakinesis) of oocytes, we observe robust TOP-2 localization on the chromosomes of all diakinesis oocytes in addition to nucleoplasmic TOP-2::GFP (Figure 1E).

To determine whether the addition of the GFP protein tag impacts the function of TOP-2 we examined the effects on embryonic viability and brood size in the *top-2::gfp* worm strain. Overall brood size of the *top-2::gfp* worm strain was slightly reduced in *top-2::gfp* worms [243 (wild type, N2) vs. 194 (*top-2::gfp*), p=0.0001, Figure 1G]. The slight reduction in brood size in *top-2::gfp* is consistent with the *top-2::3Xflag* (Avg brood=141.5) strain we generated in a previous study (Jaramillo-Lambert *et al.* 2016). The reduction in brood size could either be due to minor disruptions in TOP-2 function in mitotically dividing cells within the proliferative zone of the germ line or due to TOP-2 defects in sperm production. As we aim to use the TOP-2::GFP line to study the role of TOP-2 in male meiosis and spermatogenesis, future experiments will help resolve the two possibilities. However, embryonic viability of *top-2::gfp* worms is not significantly different from wild-type embryonic viability [99.1% (wild type) vs. 98.9% (*top-2::gfp*), p= 0.79, Figure 1H]. In addition, we monitored the TOP-2::GFP strain for a high incidence of males phenotype, which is the consequence of X-chromosome nondisjunction. X-chromosome nondisjunction in wild-type worms is rare with less than 0.3% male progeny in self-fertilizing hermaphrodites (Hodgkin, Horvitz, and Brenner 1979; Lui and Colaiácovo 2013). The percent of males produced in *top-2::gfp* was not significantly different than wild-type worms [0% (wild type) vs. 0.6% (*top-2::gfp*), p=0.06]. These results indicate that the addition of the GFP protein tag to TOP-2 is not disrupting the overall meiotic functions of this important enzyme. We conclude that the endogenous C-terminally tagged TOP-2::GFP creates a functional TOP-2 protein that is suitable for endogenous protein localization studies.

## Methods

Generation of C-terminally tagged TOP-2::GFP via CRISPR-Cas9: A C-terminally meGFP-tagged version of TOP-2 (*top-2::gfp*) was created via the direct delivery method (Paix *et al.* 2017) using *dpy-10* as a co-CRISPR marker (Arribere *et al.* 2014). The injection mix contained Cas9 protein (10 mg), *dpy-10* CRISPR RNA (crRNA) (3.2 mg), *dpy-10(cn64)* repair oligonucleotide (0.2 mg), universal trans-activating crRNA (tracrRNA) (20 mg, Dharmacon, GE Life Sciences), and a crRNA targeting the c-terminal sequence of *top-2* (GACGCGUCGUCGACUCCGACUGUUUUAGAGCUAUGCUGUUUG, 8 mg). The repair template was generated by PCR using primers containing meGFP (pAP996, Paix *et al.* 2017) and *top-2* forward (5’-TTACGACGTGGATTCAGGATCCGATTCGGATCAGCCAAAGAAGAAGAGGAGACGCGTCGT CGACTCCGACTCAGAT**TCCAAGGGAGAGGAGCTCTTCACCGG**– 3’) and *top-2* reverse sequences (5’GGAAAGAAAATAAATTAGAAACACTTAAGGAAGGTGGGAAACAAATTAATTTA**CTTGTAGAGCTCGTCCATTCCGTGG**-3’). The bold regions in the primers refer to the meGFP sequence. Edits were confirmed through PCR using a forward primer (5’-CGACAGTGATGAAGAAGCTG-3’ ) and a reverse primer (5’-GTCCTCCTTGAAGTCGATTC-3’). The crRNA was designed to have a low possibility of off-target sites using http://crispr.mit.edu. Two independent strains were created (AG330 and AG332). Strain AG332 was the focus of this study.

Live imaging was conducted by placing 10 worms on a slide with a fresh 2% agarose pad and 10 µl of anesthetic (2 mM tetramisole in M9 buffer). A glass coverslip was then placed on top of the worms and agarose pad and the space between the agarose pad and the edges of the coverslip were filled by pipetting M9 under the coverslip. Live images were taken on a Zeiss LSM880 confocal microscope using either the 40x (Figure 1A, B, C, & F) or the 63x (Figure 1D & E) objectives. Image processing and analysis was done on Fiji Is Just ImageJ (Schindelin *et al.* 2012).

Embryonic viability assays for the wild type (N2) and *top-2(av87)* [*top-2::gfp*] strains were conducted at 20°C. Both strains were grown on MYOB petri dishes inoculated with the *E. coli* (OP50). Ten L4 hermaphrodite larvae of each strain were put onto separate plates and allowed to grow and lay eggs for 16-24 h (one worm per plate). After each 24 h period, the adult (parent) worm was transferred onto a fresh plate until no additional embryos were produced. The total number of progeny (larvae and dead embryos) were counted one day after the parent was transferred. Three independent trials were conducted for each strain.

Brood sizes were determined by placing a single L4 hermaphrodite for both strains [N2 and *top-2(av87)* (*top-2::gfp*)] on a 35 mm MYOB plate spotted with *E. coli* (OP50). Each hermaphrodite was allowed to lay embryos for 24 h at 20°C. After 24 h the hermaphrodite was transferred to a fresh plate until no more embryos were produced (two additional transfers). Total brood size was calculated by adding together live larvae and dead embryos produced from a single hermaphrodite.

## Reagents

AG332 *top-2(av87)* [*top-2::gfp*] is available from the Jaramillo-Lambert Lab.

N2 is available from the CGC.
